# Alcohol consumption and its interaction with genetic variants are strongly associated with the risk of type 2 diabetes: a prospective cohort study

**DOI:** 10.1186/s12986-019-0396-x

**Published:** 2019-09-13

**Authors:** Hairong Yu, Tao Wang, Rong Zhang, Jing Yan, Feng Jiang, Shanshan Li, Weiping Jia, Cheng Hu

**Affiliations:** 1 Shanghai Diabetes Institute, Shanghai Key Laboratory of Diabetes Mellitus, Shanghai Key Clinical Center for Metabolic Diseases, Shanghai Jiao Tong University Affiliated Sixth People’s Hospital, 600 Yishan Road, Shanghai, 200233 People’s Republic of China; 2 Department of Endocrinology, The Second Affiliated Hospital of Soochow University, Suzhou, 215004 People’s Republic of China; 3 Institute for Metabolic Disease, Fengxian Central Hospital Affiliated to Southern Medical University, Shanghai, 201499 People’s Republic of China

**Keywords:** Alcohol, Genetic risk, Interaction, Insulin resistance, Beta cell function, Type 2 diabetes

## Abstract

**Background:**

Both genetic and lifestyle factors contribute to the incidence of type 2 diabetes. It yet remains controversial whether and how alcohol consumption, one of the most prevalent lifestyle habits, influences type 2 diabetes. Moreover, whether alcohol consumption interacts with genetic risk is inconclusive. Thus, we aimed to explore the effects of alcohol, genetic risk and their potential interactions on type 2 diabetes risk.

**Methods:**

The Shanghai Diabetes study (SHDS) had a total of 2546 participants with 611 incident cases of combined type 2 diabetes and impaired glucose regulation (IGR). We constructed weighted genetic risk score (GRS) for type 2 diabetes and categorized the GRS into three strata. And the homeostatic model assessment of β-cell function (HOMA-B) and insulin resistance (HOMA-IR) were calculated. Then we used logistic regression models and multiple linear regression models to examine the influence of both baseline alcohol consumption and genetic risk on blood glucose deterioration, insulin resistance (IR) and beta cell function (BC), respectively. Moreover, we investigated the interactions of alcohol intake with: (1) GRSs for type 2 diabetes, IR, BC, body mass index (BMI) and waist-to-hip ratio (WHR); and (2) each of the single nucleotide polymorphisms (SNPs) used to establish the GRSs mentioned above.

**Results:**

Alcohol consumption and higher T2D-GRS both contributed to a higher incidence rate of blood glucose deterioration [odds ratio (OR), 2.24, 95% confidence interval (CI), 1.76–2.87; OR, 1.25, 95% CI, 1.11–1.42; respectively]. Alcohol reduced insulin sensitivity and compensated by enhancing beta cell function (β = 1.98, *P* < .0001 and β = − 1.97, P < .0001 for HOMA-IR and inverse HOMA-β, respectively). T2D-GRS deteriorated insulin secretion (β = 0.10, *P* = 0.0069 for inverse HOMA-B) but not insulin sensitivity (*P* = 0.0856). Moreover, there was a significant interaction between alcohol and T2D-GRS (P_interaction_ = 0.0318), suggesting the association between alcohol and type 2 diabetes was much stronger in the lower T2D-GRS group than in the higher T2D-GRS group. And this interaction was more pronounced in men (P_interaction_ = 0.0176) than in women (P_interaction_ = 0.3285). No single SNP interacted strongly with alcohol intake.

**Conclusions/interpretation:**

Alcohol consumption strongly increased the risk of type 2 diabetes by increasing IR, especially in men with low T2D-GRS, highlighting the importance of refraining from drinking alcohol when making recommendations for healthy lifestyle habits to prevent diabetes.

## Background

Type 2 diabetes, whose development is a progress of blood glucose deterioration, is one of the most common chronic diseases worldwide. Type 2 diabetes originates from both genetic and lifestyle factors, as well as their interactions [1]. Several lifestyle factors such as smoking [2] and diet [3] have been suggested to play a role in the development of type 2 diabetes. Since alcohol is one of the most widely consumed beverages, investigating whether alcohol intake has an effect on the risk of type 2 diabetes is of great importance to public health. However, available literatures on the association between alcohol consumption and type 2 diabetes are inconsistent and controversial. The majority of the studies revealed that moderate alcohol consumption reduced the risk of type 2 diabetes, whereas high alcohol intake was associated with a higher risk [4–6]; however, the definition of the intake amount for moderate and high alcohol consumption varied in different studies. There are still a few studies indicating that alcohol consumption increases the incidence of type 2 diabetes regardless of the amount consumed [7, 8].

Both insulin resistance (IR) and impaired beta cell function (BC) are two important mechanisms in the development of type 2 diabetes. However, the effect of alcohol on insulin also remains unclear. Several cross-sectional studies have reported a negative association of alcohol consumption with beta cell function but a positive association with insulin sensitivity [9, 10]. In a prospective study in Japan [11], the researchers concluded that alcohol was positively associated with the incidence of both impaired BC and IR but showed no significant effect on type 2 diabetes.

In addition, whether alcohol consumption interacts with genetic variants remains unknown. In a more recent cohort study, both low and high intakes of alcohol increased insulin resistance but exhibited no interactions with genetic risk for IR [12].

As a result, we conducted this population-based prospective study to investigate the impact of alcohol intake and genetic risks on type 2 diabetes and to explore how they interact with each other.

## Methods

### Subjects

The Shanghai Diabetes study (SHDS) (*n* = 2546) was a community-based prospective cohort that commenced between 1998 and 2001 and enrolled 5994 subjects. There were 2546 individuals (2246 with normal glucose tolerance (NGT) and 300 with impaired glucose regulation (IGR)) who were eligible for the study after excluding those without a DNA sample for genotyping or with diagnosed type 2 diabetes, cancer, or psychiatric disturbances at baseline.

Participants were followed over a period of up to nearly 14 years (mean follow-up of 6 years). Ultimately, 2036 subjects completed the first follow-up from 2003 to 2004 and 1197 participated in the second follow-up from 2010 to 2012. To increase the sensitivity of the data, we adopted blood glucose deterioration as the positive event, which was defined as an incident development of type 2 diabetes or IGR from NGT or the development of type 2 diabetes from IGR.

Type 2 diabetes and IGR were diagnosed according to the 1999 World Health Organization definition. All subjects provided written informed consent. In addition, approval for this study was obtained from the Institutional Review Board of Shanghai Jiao Tong University Affiliated Sixth People’s Hospital.

### Alcohol intake status and biochemical measurements

After being recruited into the cohort, all individuals completed a questionnaire on their lifestyle habits, including their alcohol consumption status. The alcohol drinkers were defined as individuals who answered positively to the question “do you drink alcohol now?” or those who self-reported they had consumed liqueur, red wine, beer or any other kind of alcohol in the last year. Accordingly, those who answered negatively or did not consume alcohol in the past year were defined as nondrinkers.

Oral glucose tolerance tests (OGTTs) were performed for each individual at baseline and at the two follow-up visits to measure fasting and postprandial 2 h plasma glucose levels using the glucose oxidase method. Fasting insulin levels were also measured by radioimmunoassay (Linco Research, St. Charles, MO, USA) at baseline and at the second follow-up visit. IR and BC were assessed using the following homeostatic model assessment (HOMA) methods [13]: HOMA-derived insulin resistance index (HOMA-IR) and HOMA-derived beta cell function (HOMA-B), respectively, both of which have been used extensively in epidemiological studies [14]. The formulas were as follows [13]: HOMA-IR = fasting insulin concentration (mU/l) × fasting glucose concentration (mmol/l)/22.5; HOMA-B = 20 × fasting insulin concentration/(fasting glucose concentration-3.5).

### SNP selection, genotyping and quality-control analysis

As described elsewhere [15], we genotyped 89 SNPs in a cross-sectional Chinese population which had been reported to be associated with type 2 diabetes. From the results, we selected 40 SNPs that were significantly associated with type 2 diabetes in the Chinese population; these SNPs, including 5 SNPs associated with IR and 17 associated with BC [16, 17] (Additional file 1), were initially detected in European populations and then the detection was replicated in Chinese populations. We also selected almost all of the reported SNPs associated with body mass index (BMI) and waist-to-hip ratio (WHR) which were primarily identified in European populations and subsequently replicated in East Asian populations. After filtering those variants that were in linkage disequilibrium with each other (r^2^ > 0.8) and those that are monomorphic in the Han Chinese population, a total of 31 BMI-SNPs and 11 WHR-SNPs were genotyped [18] (Additional file 2). None of the SNPs failed the quality control tests, with call rates > 95% and concordant rates > 99%.

### GRS calculation

Given the varying effect size of different SNPs, the weighted genetic risk score (GRS) was used to represent the genetic risk of type 2 diabetes, BMI and WHR. We first calculated the weighted score by multiplying each individual’s risk allele score (0, 1, 2) by the SNP’s relative effect size (β coefficient) from previous studies conducted in East Asian or Chinese populations [15, 18] and adding the scores. Then, the value of the weighted computation was rescaled by dividing all values by the sum of the β coefficients and then multiplying the values by the total number of SNPs, thus obtaining the final weighted GRS [19]. Regarding missing data, only subjects whose data were missing > 15% of the total SNPs (that is, 6 SNPs for type 2 diabetes, 4 for BMI and 1 for WHR) were excluded. The weighted GRSs of the remaining individuals with missing data were standardized to those of individuals with complete data. The computational formula was as follows: standardized weighted GRS = [(weighted GRS based on non-missing genotypes)/ (number of non-missing genotypes)] × (the total number of the SNPs).

In addition, we created an unweighted GRS for BC and IR using the SNPs already used in the T2D-GRS model. Briefly, the unweighted GRS was calculated by summing the number of risk alleles of each SNP. We chose to leave these GRSs unweighted since the SNPs were identified based on different measurements of both insulin secretion and insulin resistance, making it difficult to give a single weight to each SNP.

### Statistical analysis

GRSs were categorized into three strata, namely, low, intermediate and high tertiles. Within each of the six strata defined by the GRS and alcohol intake, we calculated incidence by dividing the number of incident cases by the corresponding person time (incidence per 10,000 person-years of follow-up). We used logistic regression models to test the role of genetic and alcohol factors and their interactions on the incidence of blood glucose deterioration. Multiple linear regression analysis was carried out to test the association of alcohol and the GRS with BC and IR. In these linear regression analyses, HOMA-IR and inverse HOMA-B (1/HOMA-B) were first multiplied by 10 and log_10_ transformed before they were subsequently converted to standardized z scores. The change in IR was calculated using the log_10_ transformed HOMA-IR from the second follow-up visit minus the log_10_ transformed HOMA-IR at baseline. The change in BC was determined using the same method.

To further explore how alcohol and GRSs interacted with each other, we examined the associations of alcohol with the incidence of glucose deterioration, IR and beta cell function in each GRS tertile. In addition, analysis was performed to evaluate the role of GRSs within alcohol drinker and nondrinker groups. We also examined interactions separately by sex. The interactions between alcohol intake and each SNP were analysed.

All analyses were adjusted for covariates such as age, sex, BMI and smoking status at baseline. Baseline BC and IR were also adjusted when analysing the data from the second follow-up period. All analyses were conducted using PLINK1.9 [20] and SAS (version 8.0; SAS Institute, Cary, NC, USA). *P* < 0.05 was considered statistically significant.

## Results

### Baseline characteristics

Table 1 shows the baseline characteristics of the individuals in the SHDS cohort. With the exception of differences in fasting glucose (*P* = 0.0006), there were no significant differences in baseline characteristics across the T2D-GRS tertiles.

**Table 1:** Baseline characteristics of individuals stratified into three tertiles by weighted T2D-GRS

Variables		Weighted type 2 diabetes GRS			*P* value
	Overall (n = 2546)	Low (*n* = 848)	Intermediate (*n* = 849)	High (*n* = 849)	
Age (years)	54.17 ± 14.73	54.25 ± 14.99	54.46 ± 14.26	53.79 ± 14.93	0.6335
BMI (kg/m2)	24.94 ± 3.46	23.92 ± 3.40	24.08 ± 3.56	23.83 ± 3.43	0.3491
Man (n, %)	1029 (47.49)	356 (41.98)	332 (39.1)	341 (40.16)	0.4745
Smoking (n, %)	494 (22.85)	176 (24.55)	144 (19.81)	174 (24.23)	0.0559
Drinking (n, %)	1538 (71.2)	519 (72.49)	514 (70.7)	505 (70.43)	0.6467
Sugar intake (n, %)	1199 (52.91)	396 (53.23)	384 (50)	419 (55.57)	0.0915
Fasting plasma glucose (mmol/l)	4.97 ± 0.53	4.91 ± 0.50	4.98 ± 0.55	5.01 ± 0.54	0.0006
2 h plasma glucose (mmol/l)	5.68 ± 1.62	5.63 ± 1.53	5.68 ± 1.63	5.74 ± 1.70	0.3922
Fasting insulin (mU/l)	6.97 (4.62, 10.04)	6.82 (4.62, 9.91)	7.19 (4.69, 10.12)	6.95 (4.57, 10.12)	0.2754
2 h insulin (mU/l)	39.12 (24.09, 59.86)	39.59 (24.69, 59.86)	39.11 (24.20, 58.97)	38.70 (23.72, 60.93)	0.9809
HOMA-IR	1.53 (0.99, 2.26)	1.48 (0.97, 2.20)	1.56 (1.00, 2.35)	1.56 (1.01, 2.29)	0.1602
HOMA-B	99.55 (67.54, 144.76)	100.95 (69.44, 145.37)	101.69 (68.63, 146.91)	97.04 (63.38, 141.82)	0.1585
Incident cases (n)	611	168	207	236	0.0012

Low: weighted T2D-GRS < 43.0; Intermediate: 43.0 ≤ weighted T2D-GRS < 46.7; High: weighted T2D-GRS ≥ 46.7

Data are shown as the mean ± SD, median (interquartile range) or n (%)

Comparisons were done using ANOVA test

### Associations of GRSs with type 2 diabetes

**Fig. 1:**
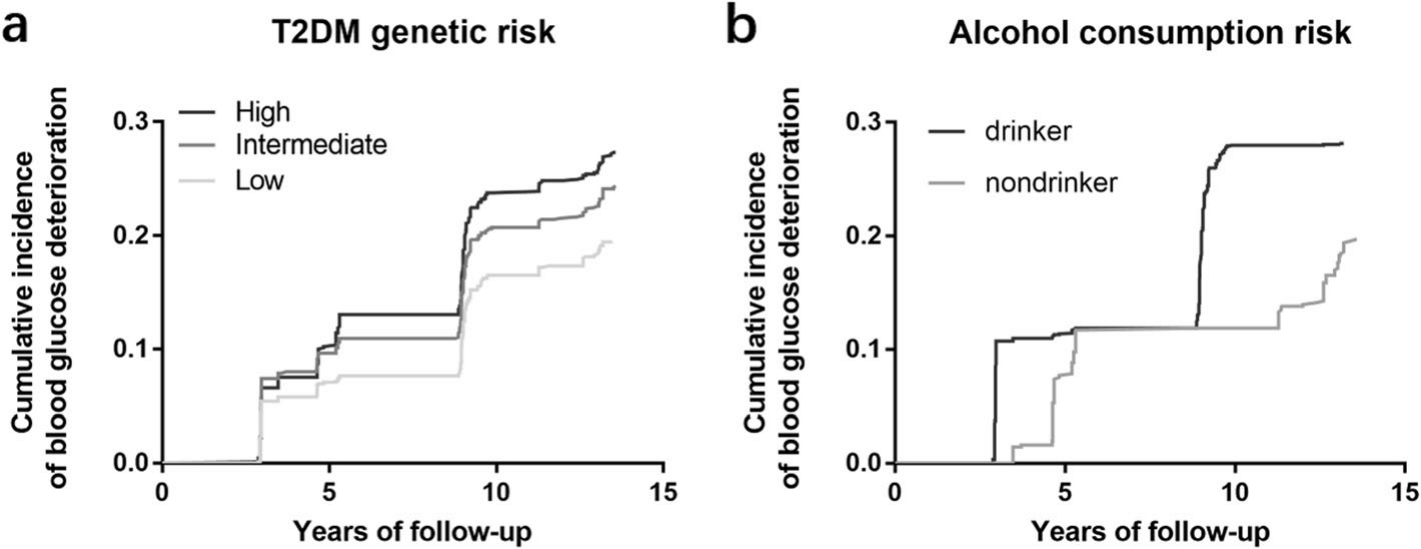
Incidence of combined type 2 diabetes and IGR. Shown are cumulative incidence of combined type 2 diabetes and IGR, according to type 2 genetic risk (**a**) and alcohol consumption (**b**), respectively, during the entire follow-up period

All GRSs with the exception of the BMI-GRS and the BC-GRS were significantly associated with the deterioration of blood glucose. However, only association between alcohol and T2D-GRS remained significant after multiple testing. Among the GRSs, the T2D-GRS had the strongest effect [odds ratio (OR), 1.25, 95% confidence interval (CI), 1.11–1.42, P for trend = 0.0004) (Table 2). The cumulative incidence of combined type 2 diabetes and IGR increased significantly across the tertiles of T2D-GRS (Fig. 1). Multiple linear regression revealed that the T2D-GRS mainly affected BC (β = 0.10, *P* = 0.0069 for inverse HOMA-B) rather than IR (*P* = 0.0856 for HOMA-IR) (Additional file 3).

**Table 2:** Effect of alcohol and GRSs on blood glucose deterioration

	Overall		Men		Women	
	OR (95% CI)	*P*-value	OR (95% CI)	*P*-value	OR (95% CI)	*P*-value
Alcohol	2.24 (1.76, 2.87)	<.0001*	1.84 (1.20,2.84)	**0.0055**	2.47 (1.84, 3.32)	0.0032*
T2D-GRS	1.25 (1.11, 1.42)	0.0004*	1.24 (1.03, 1.51)	**0.0269**	1.26 (1.07, 1.49)	**0.0062**
BC-GRS	1.04 (0.99, 1.08)	0.0565	1.05 (0.99, 1.11)	0.1384	1.03 (0.98, 1.08)	0.2151
IR-GRS	1.09 (1.01, 1.17)	**0.0308**	1.17 (1.04, 1.32)	**0.009**	1.03 (0.94, 1.14)	0.5164
WHR-GRS	1.14 (1.00, 1.30)	**0.0439**	1.30 (1.06, 1.60)	**0.0113**	1.05 (0.88,1.24)	0.6032
BMI-GRS	0.92 (0.81, 1.04)	0.1973	0.95 (0.78, 1.15)	0.5693	0.90 (0.76, 1.07)	0.232

Data are shown as odds ratio and 95% confidence intervals

Covariates: age, sex, smoking, and BMI

*P* values <0.05 were shown in bold. * indicates significance after Bonferroni correction

### Associations of alcohol consumption with type 2 diabetes

Regarding the effect of alcohol consumption, alcohol drinkers had a higher risk of blood glucose deterioration than nondrinkers (OR, 2.24, 95% CI, 1.76–2.87) both in men (*P* = 0.0055) and women (*P* = 0.0032) (Table 2), indicating that alcohol was a strong risk factor of blood glucose deterioration. As shown in Fig. 1, the cumulative incidence of blood glucose deterioration was much higher in alcohol drinkers than in nondrinkers. There was a significant difference in incidence between alcohol drinkers and nondrinkers at the end of follow-up (*P* < .0001). Alcohol consumption was positively associated with IR (β = 1.97, P < .0001 for HOMA-IR) and compensated by enhancing beta cell function (β = − 1.97, P < .0001 for inverse HOMA-B) (Additional file 3).

**Fig. 2:**
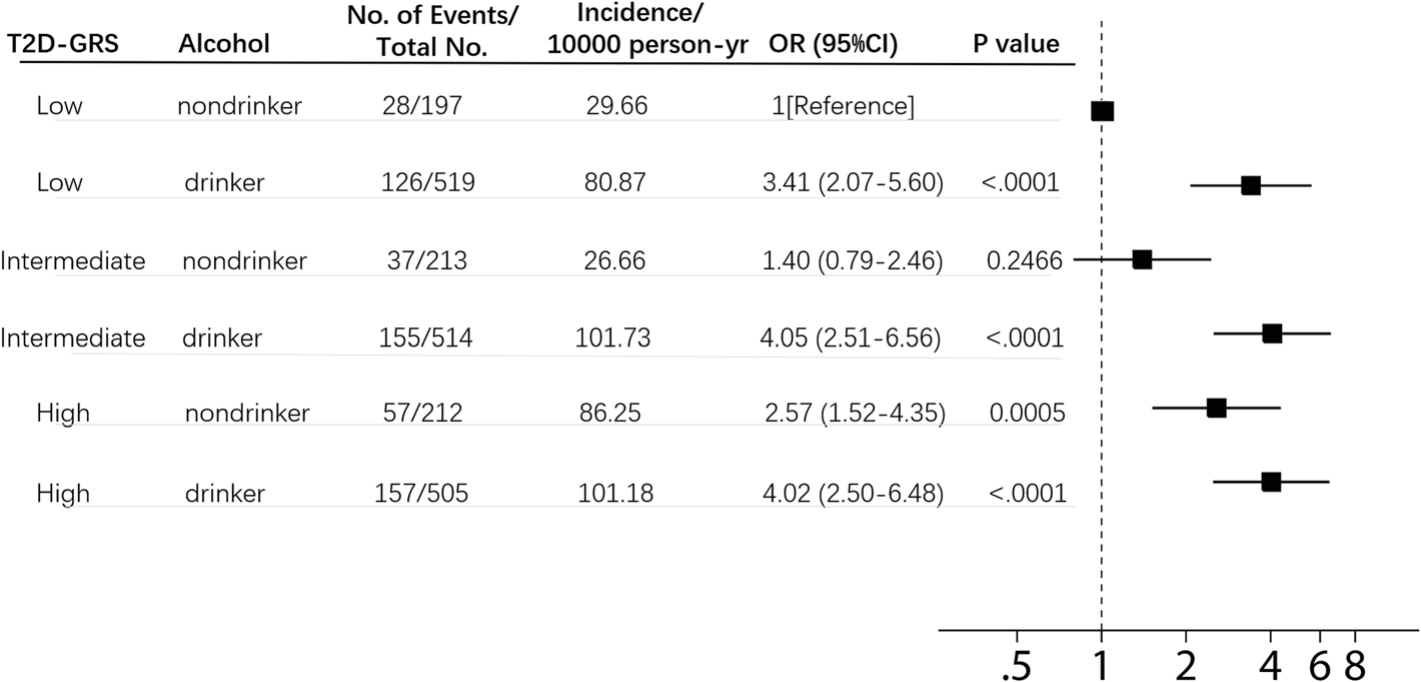
Risk of blood glucose deterioration. Shown are adjusted odds ratios with 95% confidence intervals for blood glucose deterioration, according to genetic risk and alcohol consumption. In these comparisons, alcohol nondrinkers with low genetic risk served as the reference group

Within each stratum of type 2 diabetes genetic risk, alcohol consumption was a powerful risk factor of diabetic events. The effect of alcohol on individuals with a high genetic risk seemed not to be as strong as the effect on individuals with a low genetic risk. Similarly, the T2D-GRS seemed to have a weaker effect on the alcohol drinker group than on the nondrinker group (Fig. 2).

### Interactions of genetic risk and alcohol consumption and sex differences

There was a significant interaction between the T2D-GRS and alcohol consumption in the entire cohort (OR_interaction_, 0.72, 95% CI, 0.54–0.97, P_interaction_ = 0.0318) (Additional file 4). However, this interaction did not survive after multiple testing correction. The association of alcohol with type 2 diabetes incidence was strongest in the low T2D-GRS group and weakest in the high GRS group (Table 3). Specifically, in the low T2D-GRS group, alcohol drinkers had a greater than three times higher risk of glucose deterioration than nondrinkers (OR, 3.33; 95% CI, 2.04–5.43), which corresponded to an increase in the incidence of blood glucose deterioration from 29.66 to 80.87 cases per 10,000 person-years (Fig. 2). Nevertheless, among individuals with a high genetic risk, alcohol drinkers had only a 50% higher risk (OR, 1.53; 95% CI, 1.04–2.23), with the incidence increasing from 86.25 to 101.18 cases per 10,000 person-years. Accordingly, the association of the T2D-GRS with glucose deterioration was more significant among alcohol nondrinkers (OR, 1.62; 95% CI, 1.24–2.10) than drinkers (OR, 1.1,17; 95% CI, 1.01–1.35). This interaction of alcohol consumption with the T2D-GRS remained in the men (OR_interaction_, 0.51, 95%CI, 0.30–0.89, P_interaction_ = 0.0176) but not in the women (P_interaction_ = 0.3285) when examined separately by sex (Additional file 4). In the subgroup of men, the interactions remained the same as in the entire cohort. However, when using the continuous T2D-GRS, we found that there was no significant interaction between alcohol drinking and T2D-GRS (*P* = 0.1079).

**Table 3:** Effect of alcohol on blood glucose deterioration incidence by tertile of T2D-GRS

	Overall		Men	
T2D-GRS	OR (95% CI)	*p*-value	OR (95% CI)	*p*-value
Low	3.33 (2.034, 5.43)	**<.0001**	6.59 (2.21, 19.70)	**0.0007**
Intermediate	2.63 (1.70, 4.07)	**<.0001**	1.39 (0.66, 2.92)	0.3808
High	1.53 (1.04, 2.23)	**0.0293**	1.10 (0.57, 2.12)	**0.7802**

Data are shown as odds ratios and 95% confidence intervals.

Covariates: age, smoking, sex and BMI.

Low: T2D-GRS < 43.0; Intermediate: 43.0 ≤ T2D-GRS< 46.7; High: T2D-GRS ≥ 46.7

*P* values <0.05 were shown in bold

We observed a significant interaction of the BC-GRS with alcohol in terms of type 2 diabetes risk only in the subgroup of men (OR_interaction_, 0.58, 95% CI, 0.34–0.98, P_interaction_ = 0.0423) (Additional file 4). The trend was the same as that detected for the T2D-GRS in that the association of alcohol with glucose deterioration was strongest in the low genetic risk group (Additional file 5), and the effect of the BC-GRS was stronger among alcohol nondrinkers. However, no evidence of an interaction between the IR-GRS and alcohol was detected. The WHR-GRS and the BMI-GRS showed no significant interactions with alcohol consumption either in the entire cohort or in the subgroup of men and women.

In addition, a significant interaction of the BC-GRS and alcohol in relation to beta cell function was observed in the subgroup of men (β_interaction_ = − 0.39, P_interaction_ = 0.0299 for inverse HOMA-B and β_interaction_ = 0.46, P_interaction_ = 0.0105 for HOMA-B change) (Additional file 6). The differences in inverse HOMA-B between alcohol drinkers and nondrinkers amounted to − 1.30 (SE = 0.31) in the lowest BC-GRS group and − 2.07 (SE = 0.22) in the highest BC-GRS group (Additional file 5). Similarly, the difference in HOMA-B change from baseline to the second follow-up was 1.10 (SE = 0.28) and 2.03 (SE = 0.22) in the lowest and highest tertiles of genetic risk, respectively. Although the interaction of the IR-GRS with alcohol in relation to HOMA-IR was significant in men (P_interaction_ = 0.0278), there was no interaction for the change in HOMA-IR (P_interaction_ = 0.1606).

None of the 82 SNPs included in the GRSs (40 for type 2 diabetes, 31 for BMI and 11 for WHR) showed a significant interaction with alcohol intake in relation to type 2 diabetes after correction of multiple testing (Additional file 1).

## Discussion

In this prospective community-based SHDS study, we concluded that alcohol played an important role in the development of type 2 diabetes by increasing insulin resistance. Type 2 diabetes risk was approximately 1.5-fold higher for alcohol drinkers than for nondrinkers. Alcohol consumption interacted with type 2 diabetes genetic risk. Although this interaction remained insignificant after multiple testing correction as multiple testing may be too strict to make interactions survive, it suggested that the interaction was very likely to exist. Specifically, the association of alcohol and type 2 diabetes was much stronger in the lower T2D-GRS group than in the higher T2D-GRS group. Furthermore, our results showed that this interaction was mainly driven by the genetic risk for impaired beta cell function as opposed to that for IR and was more significant in men than in women.

The empirical evidence regarding the role of alcohol and its interaction with genetic risk in relation to type 2 diabetes remains limited. To the best of our knowledge, our study is the first prospective Chinese study to explore the interaction between alcohol and genetic risk in relation to type 2 diabetes, and our study further studied two well-known important mechanisms of the development of diabetes, namely, insulin resistance and impaired beta cell function. As shown in our study, alcohol increased type 2 diabetes risk by extremely increasing IR and accordingly increasing beta cell function for compensation. In previous studies using HOMA-IR as the IR index, some researchers found a significant inverse dose-response association between alcohol consumption and mean HOMA-IR [21]; some reported a U-shaped association [22]; and some results corresponded with our study and showed that alcohol consumption was positively associated with IR [11]. Therefore, further prospective studies are needed to confirm the association between alcohol consumption and IR. In our study, alcohol interacted with the T2D-GRS in that alcohol increased type 2 diabetes risk in each T2D-GRS tertile, especially in the lowest one. We proposed that alcohol was a powerful risk factor of type 2 diabetes. Alcohol drinkers thus had a much higher risk than nondrinkers among individuals with a low genetic risk for diabetes, whereas in the high T2D-GRS group, individuals were already at high risk for diabetes due to their high genetic risk score; therefore, the impact of alcohol was relatively weakened.

Our study also further clarified that the interaction between alcohol and the T2D-GRS on diabetes worked principally through the BC-GRS to influence beta cell function. Few studies have focused on the interaction of alcohol with the genetic risk related to beta cell function. A Korean study reported that alcohol interacted with BC-related genetic variants but lowered insulin secretion [23]. Further well-powered studies investigating potential interactions are highly warranted.

The strengths of our study include the use of a prospective cohort study; the use of a total number of 40 SNPs to define type 2 diabetes genetic risk, all of which had already been validated in the Chinese population; and the use of the genetic dissection of the pathophysiological process to further explore the underlying mechanism of type 2 diabetes.

There were also several limitations. First, the number of participants lost to the second follow-up was relatively high, which may have been due to the migration away from the community as Shanghai has rapidly urbanized in the last decades. In addition, among lost individuals in the second follow-up, there was no obvious difference in the proportions of tertiles of T2D-GRS in drinkers and nondrinkers (*P* = 0.1773), which suggests the loss was random. Therefore, we speculated that the influence of loss to follow-up on our main finding that there is a stronger effect of T2D-GRS on T2D risk in non-drinkers compared to effects in drinkers would be limited. Second, self-reported alcohol consumption questionnaires are subject to error due to reporting errors on the part of the subjects; Data on amount and frequency of alcohol consumption and specific alcohol beverage types were missing; And whether alcohol consumption pattern changed during the entire follow-up period was not assessed. Third, the GRS explains only a fraction of the expected heritability and hence likely results in low statistical power to assess interactions with individual SNPs. And the power value of the interaction analysis for alcohol and T2D-GRS in women was 0.69. Considering the relatively low prevalence in alcohol drinking in women, the sample size should be enlarged. In future studies, it will be of great importance to utilize less subjective measures of alcohol consumption status and intake amount, to replicate the investigation in another larger prospective study and to further explore the role of alcohol in the underlying mechanisms of diabetes.

## Conclusions

In conclusion, our work is relevant as the contribution of alcohol to the development of type 2 diabetes remains unclear. Our prospective study revealed that alcohol was a strong risk factor of blood glucose deterioration mainly by increasing IR. Besides, alcohol interacted with genetic risk for T2D. Alcohol consumers had much higher risk of T2D among individuals with a low genetic risk than those with a high risk. Therefore, it is of great significance to advocate the cessation of alcohol when making recommendations for healthy lifestyle habits to prevent diabetes.

## Supplementary information

**Additional file 1.** SNPs used for T2D genetic risk score.

**Additional file 2.** SNPs used for BMI and WHR genetic risk score.

**Additional file 3.** Effect of alcohol and GRSs on insulin resistance and beta cell function.

**Additional file 4.** Interaction of T2D, BC, IR, BMI and WHR gentic risk score with alcohol on blood glucose deterioration.

**Additional file 5.** Effect of alcohol on blood glucose deterioration and insulin secretion by tertile of BC-GRS in the subgroup of men.

**Additional file 6.** Interaction of T2D, BC and IR GRS with alcohol on insulin resistance and insulin secretion.

## Data Availability

The datasets analysed for our study are not publicly available due to ethical restrictions related to the consent given by subjects at the time of study commencement. Data are however available from the corresponding author upon reasonable request and with permission of the Institutional Review Board of Shanghai Jiao Tong University Affiliated Sixth People’s Hospital.

## Abbreviations

BC: Beta cell function
BMI: Body mass index
GRS: Genetic risk score
IGR: Impaired glucose regulation
IR: Insulin resistance
NGT: Normal glucose tolerance
SNP: Single nucleotide polymorphism
T2D: Type 2 diabetes
WHR: Waist-to-hip ratio
